# Quantitative Super-Resolution Microscopy to Assess Adhesion of Neuronal Cells on Single-Layer Graphene Substrates

**DOI:** 10.3390/membranes11110878

**Published:** 2021-11-15

**Authors:** Silvia Scalisi, Francesca Pennacchietti, Sandeep Keshavan, Nathan D. Derr, Alberto Diaspro, Dario Pisignano, Agnieszka Pierzynska-Mach, Silvia Dante, Francesca Cella Zanacchi

**Affiliations:** 1 Nanoscopy and NIC@IIT, Istituto Italiano di Tecnologia, 16152 Genoa, Italy; silvia.scalisi-1@unitn.it (S.S.); francesca.pennacchietti@scilifelab.se (F.P.); Alberto.Diaspro@iit.it (A.D.); agnieszka.pierzynska-mach@iit.it (A.P.-M.); 2 DIFILAB, Department of Physics, University of Genoa, 16146 Genoa, Italy; 3 Department of Cellular, Computational and Integrative Biology (CIBIO), University of Trento, 38122 Trento, Italy; 4 Materials Characterization Facility, Istituto Italiano di Tecnologia, 16163 Genoa, Italy; sandeep.keshavan@unifr.ch; 5 Center for Microscopy and Imaging & Department of Biological Sciences, Smith College, 44 College Lane, Northampton, MA 01063, USA; nderr@smith.edu; 6 Physics Department ‘E. Fermi’, University of Pisa, 56127 Pisa, Italy; dario.pisignano@unipi.it; 7 NEST, Istituto Nanoscienze-CNR, 56126 Pisa, Italy

**Keywords:** biophysics, super-resolution microscopy, graphene, adhesion complexes, single molecule localization microscopy

## Abstract

Single Layer Graphene (SLG) has emerged as a critically important nanomaterial due to its unique optical and electrical properties and has become a potential candidate for biomedical applications, biosensors, and tissue engineering. Due to its intrinsic 2D nature, SLG is an ideal surface for the development of large-area biosensors and, due to its biocompatibility, can be easily exploited as a substrate for cell growth. The cellular response to SLG has been addressed in different studies with high cellular affinity for graphene often detected. Still, little is known about the molecular mechanism that drives/regulates the cellular adhesion and migration on SLG and SLG-coated interfaces with respect to other substrates**.** Within this scenario, we used quantitative super-resolution microscopy based on single-molecule localization to study the molecular distribution of adhesion proteins at the nanoscale level in cells growing on SLG and glass. In order to reveal the molecular mechanisms underlying the higher affinity of biological samples on SLG, we exploited stochastic optical reconstruction microscopy (STORM) imaging and cluster analysis, quantifying the super-resolution localization of the adhesion protein vinculin in neurons and clearly highlighting substrate-related correlations. Additionally, a comparison with an epithelial cell line (Chinese Hamster Ovary) revealed a cell dependent mechanism of interaction with SLG.

## 1. Introduction

In recent years, the rise of a new family of carbon-based nanomaterials has attracted increasing attention in the scientific community. Since its discovery [1], graphene has emerged as a building block of a promising nano-platform with enormous potential for biomedical engineering, translational medicine, and biotechnology [2,3]. Due to its chemical, physical, and mechanical properties, graphene and its derivatives are highly promising candidates for biosensors [4,5], tissue engineering [6,7,8,9], tissue scaffolding [10,11], gene therapy [12,13], drug delivery [14,15], and bioimaging probes [16,17,18,19]. However, the employment of graphene-related nanomaterials in a biological framework requires a detailed characterization and understanding of the effects induced by the material interaction with different living systems.

## 2. Adhesion and Proliferation of Neurons on Graphene

Due to its high transmittance and conductivity, graphene may be especially suited for biomedical applications related to neurons [20]. Indeed, neuronal functions are strongly based on electrical activity. The effects of graphene on neurons have been extensively studied, highlighting excellent compatibility with neuronal cells, as well as enhanced cellular growth and vitality compared to conventional culture substrates [21,22,23,24]. Among the different types of graphene [3], SLG grown by chemical vapor deposition is the most suitable for the development of biosensing architectures given the ease with which it can be used to functionalize other surfaces and given the possibility of its being processed by microfabrication methods [25,26]. For instance, recent studies propose SLG as a substrate for growing large-area patterned neuronal networks [27,28]. In particular, patterned surfaces of SLG are shown to promote ordered neuronal growth and preferential adhesion [28]. Enhanced adhesion to SLG by other cell types, like the epithelial Chinese hamster ovary (CHO), was also reported, although with varied response [29]. These findings indicate that the interplay with different surfaces is a cell-type-dependent mechanism. The cell response may be induced by several factors, ranging from the different characteristics of their membranes to differences in the specific cell functionality. Still, the molecular mechanisms that drive the preferential cell adhesion and migration on different substrates are partially unknown.

Mechanotransduction and adhesion play a primary role in cellular differentiation, migration, and proliferation. In particular, focal adhesions (FAs), macromolecular assemblies connecting the intracellular actin network with the extracellular matrix, transmit mechanical forces and signals linking the membrane to the cytoskeleton [30]. Mature FAs are axially separated in multiple functional nanodomains and composed of three distinct functional layers (i.e., integrin signaling layer, force-transduction layer, and actin regulatory layer). FAs consist of large complexes of transmembrane integrins whose intracellular domain binds to the cytoskeleton through adapter proteins, such as talin, α-actinin, paxillin, vinculin, and tensin. In mature FAs, vinculin acts as a **‘**molecular clutch’ to modulate the mechanical force transmission from the membrane-bound integrins to cytoplasmic F-actin [31]. With a focus on vinculin, recent studies show a correlation of the focal adhesion protein distribution in response to the different substrate stiffness [32,33].

Within this scenario, a better understanding of the molecular mechanism underlying cell migration on graphene implies a need for a quantitative study of the nanoscale distribution of vinculin in FAs. Super-resolution microscopy and single-molecule localization microscopies [34] (SMLMs) are powerful tools to study FAs [35] and to unveil their organization at the nanoscale level. In the past, two-color photo-activatable localization microscopy (PALM) demonstrated colocalization of vinculin and paxillin, showing that they form nano-aggregates [36], whereas talin plays a central role in organizing the focal adhesion strata [37]. Furthermore, new advances in quantitative super-resolution microscopy and the development of novel clustering algorithms [38,39] make single-molecule localization microscopy a suitable quantitative tool [40,41,42] for FA protein characterization [43]. In this work, we use quantitative super-resolution, based on stochastic optical reconstruction microscopy (STORM) and cluster analysis, to study the vinculin distribution in mammalian cell lines (CHO) and primary neurons, shedding new light on the molecular mechanisms behind the preferential growth of cells on graphene substrates.

## 3. Results

### 3.1. Influence of the Substrate on Quantitative SR

To characterize the focal adhesions of seeded cells, we used single-molecule localization based super-resolution microscopy. This method allowed us to quantitatively compare the vinculin distribution between glass and graphene. Cells were grown under standard conditions and seeded on different substrates coated with poly-D-lysine. Cells were fixed and immuno-stained for fluorescence super-resolution microscopy (Materials and Methods). We used stochastic optical reconstruction microscopy (STORM) and cluster analysis to identify vinculin clusters and characterize the number of localized molecules within each cluster.

**Figure 1:**
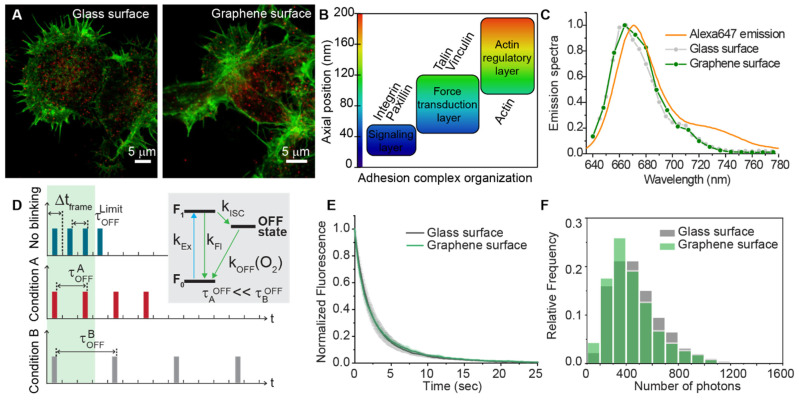
Substrate influence on single molecule detection. (**A**) Confocal imaging of DIV1 hippocampal neurons. Vinculin labeled with AlexaFluor647 (red), and F-actin stained with AlexaFluor488 (green). Scale bars: 5 µm. (**B**) FA protein organization into three functional layers and their distances from the substrate: vinculin resides within the intermediate force transduction layer. (**C**) Emission spectrum of vinculin-AlexaFluor647 on different substrates (glass and graphene) and theoretical Alexa 647 spectrum. (**D**) Scheme of the temporal evolution of the signal in a STORM recording, according to the ON/OFF ratio. Simplified sketch of the fluorophore photocycle (all the processes that influence the OFF state are grouped in a general OFF state with a global k_OFF_). Three different cases are compared: sparse regime cannot be reached (i.e., no blinking) and sparse regime with short (condition A) and long (condition B) off time/residency, respectively. The green region represents the initial part of the STORM recordings, in which most of the molecules are pushed into the dark state to reach the required molecular sparsity. According to the characteristic on/off time, a different distribution of events in time will be registered, influencing the quantitative information extractable from the single-molecule recordings. (**E**) Decay of fluorescence upon 647 nm illumination on graphene and glass surface, and (**F**) number of emitted photons.

Graphene has been proven to influence the fluorophores photo-physics [44,45]. A proper investigation of the adhesion properties using STORM requires taking into account the possible influence of graphene on the probe’s fluorescence emission, in order to avoid artifacts and the consequent wrong estimation in the quantitative analysis. To control for potential photo-physical interactions of the graphene substrate and the fluorophores used in our study, we first accessed the influence of the substrate on the fluorophore photophysics (Figure 1).

Confocal imaging of neurons grown on glass and graphene (Figure 1A) showed the distribution of vinculin (stained with Alexa647, red channel) and actin (stained with Alexa488, green channel) after one day in vitro (DIV1). Vinculin belongs to the force transduction layer (Figure 1B), and its distance (approximately 35–90 nm) from the substrate is enough to ensure a negligible graphene-induced quenching [44]. Here, the emission spectra of vinculin-Alexa 647 (Figure 1C) measured both on glass and graphene did not show any substantial difference.

Another possible aspect that the graphene monolayer could influence is the temporal evolution of the signal and single-molecule blinking dynamics (Figure 1D). Indeed, any alteration on the rates of the ON/OFF photoswitching mechanism (Figure 1D inset) will result in different initial conditions of the measurement and time evolution of the signal, compromising the possibility of comparing graphene and glass substrate quantitatively. In other words, if the time of residence in the OFF state is too short, all the events will be hidden in the initial step of the STORM recording, when all the molecules are pushed to the OFF state to reach the sparse regime (Figure 1D, green shadow in the left). Moreover, different kinetics would also result in a different number of events for a given fixed observation time, a difference that would alter density estimation in different substrates. Nevertheless, a comparison of the rate (k_OFF_) did not show a significant difference for the two surfaces (Figure 1E) and the average number of photons detected (Figure 1F) during the single-molecule recording was kept constant. These observations proved that the photo cycle is not appreciably affected by the presence of the graphene monolayer. This assessment assures suitable conditions for quantitative single-molecule localization to estimate vinculin’s local density and clustering degree in the focal adhesion points.

The negligible effect of graphene on the single-molecule blinking was further confirmed by measuring the number of localized events on nanostructured calibration standards (DNA origami) functionalized with a controlled number of fluorophores [46]. We acquired the single-molecule signal from full DNA origami structures, which were labeled with 86 fluorophores, adsorbed on glass and graphene (SLG) substrates coated with poly-D-lysine. A similar number of localization events was observed for full DNA origami structures on SLG and glass (Supplementary Figure S1), confirming the minimal effect of the substrate on the fluorophores photophysics. These controls conclude that proper vinculin quantification through single-molecule localization is not impaired by the different substrates since quenching effects and modifications of the fluorophore photo-cycle can be neglected.

### 3.2. Quantification of Vinculin in CHO Cells

**Figure 2:**
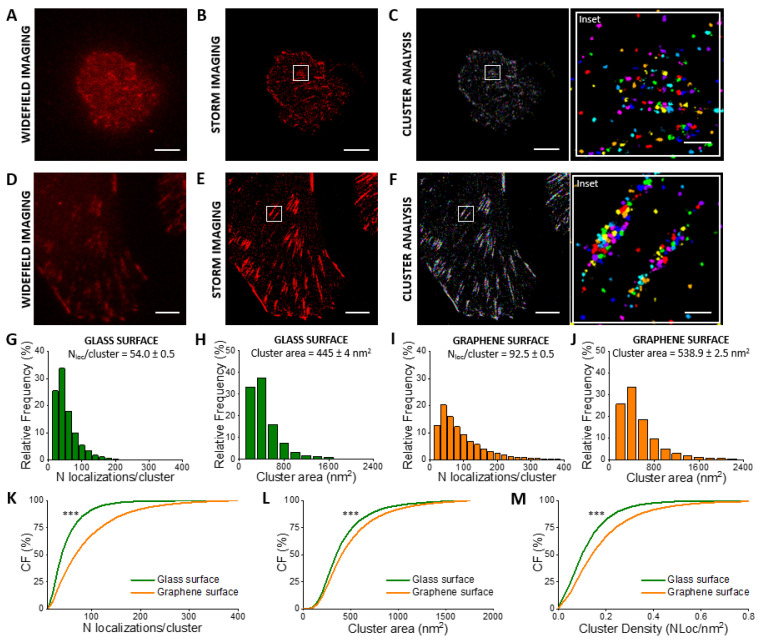
Quantitative super-resolution imaging of vinculin in CHO cells. Widefield imaging (**A**), STORM imaging (**B**), and clustering (**C**) of vinculin immuno-stained with AF405/AF647 in cells seeded on glass. Widefield imaging (**D**), STORM imaging (**E**), and clustering (**F**) of vinculin immuno-stained with AF405/AF647 in cells seeded on SLG. Frequency distribution of localizations/cluster and cluster area corresponding to cells on glass (**G**,**H**) and on SLG (**I**,**J**). The cumulative distribution shows a 71% increased average number of localized events (**K**) for vinculin in cells adhered on SLG (92.5 ± 0.5 N_loc_/cluster) compared to cells adhered on glass (54.0 ± 0.5 N_loc_***/***cluster). The cumulative distribution (CF = cumulative frequency) shows a 21% increased average cluster area (**L**) in cells adhered on SLG (445 ± 4 nm^2^) compared to cells adhered on glass (538.9 ± 2.5 nm^2^). Cumulative distribution shows a 38% increased average density (**M**) in cells adhered on SLG (0.205 ± 0.001 N_loc_/nm^2^) compared to cells adhered on glass (0.148 ± 0.001 N_loc_/nm^2^). The total number of analyzed clusters is N_glass_ = 5532 and N_graphene_ = 21032 for glass and graphene substrates, respectively. *n* > 3 independent experiments. Kolmogorov–Smirnov Statistical test: ns nonsignificant, *** *p* < 0.001. Scale bar = 5 μm. Scale bar inset = 500 nm.

To assess the feasibility of our methodological approach, we firstly imaged a well-characterized and robust cell line. We chose CHO cells (Materials and Methods), previously employed to quantify cell adhesion forces by single-cell force spectroscopy [29]. We used STORM imaging to acquire super-resolution images of vinculin in DIV2 CHO cells at the same time interval we had used previously [29]. STORM images show morphological differences between the cells adhered to glass (Figure 2A,B) and SLG (Figure 2D,E). Cells on glass are smaller and more rounded than cells on graphene that are flatter and bigger. Moreover, on SLG, vinculin forms macromolecular assemblies organized in elongated structures (FAs), absent in cells seeded on glass.

We segmented the vinculin clusters using a previously developed distance-based clustering algorithm [47] (Figure 2C,F, insets), and we quantified the number of localizations per vinculin cluster (Figure 2G,I) and the cluster area (Figure 2H,J). As shown in Figure 2K, for cells grown on graphene, the number of vinculin localizations per cluster (92.5 ± 0.5 N_loc_/cluster) significantly increased (71%) compared to cells grown on the glass substrate (54.0 ± 0.5 N_loc_/cluster). Furthermore, we observed a 21% increase of the mean cluster’s area (Figure 2L) and a 38% increase in the corresponding average density (Figure 2M).

These results are in accordance with our previous results [29], in which a much higher adhesion force of CHO cells on SLG compared to glass was measured by AFM single-cell-force spectroscopy. The higher cluster density of vinculin on SLG indeed explains the higher work to detach CHO cells from SLG than glass surfaces measured in that investigation.

### 3.3. Quantification of Vinculin in Neurons

We further studied the adhesion process in neurons, as they represent an attractive application of growing cells on graphene, thanks to the possibility to create geometrically ordered neural networks for biomedical applications.

We performed super-resolution STORM imaging of vinculin on embryonal rat neurons at different growing stages (DIV1 and DIV3, respectively).

**Figure 3:**
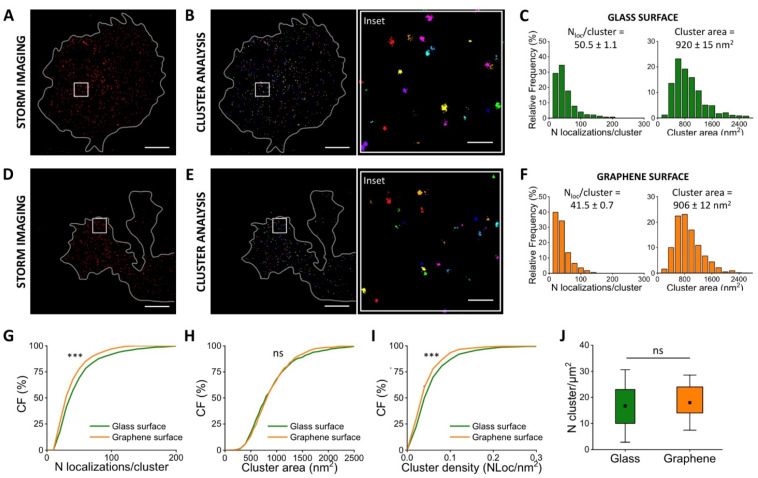
Quantitative super-resolution imaging of vinculin in neurons (DIV1). STORM imaging (**A**) and cluster analysis (**B**) of vinculin immuno-stained with A405/A647 in neurons seeded on glass. Frequency distribution of localizations/cluster and cluster area measured in cells seeded on glass (**C**). STORM imaging (**D**) and clustering (**E**) of vinculin immuno-stained with A405/A647 in neurons seeded on SLG. Frequency distribution of localizations and cluster area in cells grown on SLG (**F**). The cumulative distribution shows a 22% decrease in the average number of localized events (**G**) for vinculin-imaged in cells adhered on SLG (41.5 ± 0.7 N_loc_***/***cluster) and on glass (50.5 ± 1.1 N_loc_***/***cluster). Cumulative distribution of the cluster area (**H**) doesn’t show significant differences in cells adhered on SLG (906 ± 12 nm^2^) compared to cells adhered on glass (920 ± 15 nm^2^). The cumulative distribution shows a 21% decrease in average density (**I**) in cells adhered on SLG (0.053 ± 0.001 N_loc_/nm^2^) compared to cells adhered on glass (0.064 ± 0.002 N_loc_/nm^2^). The average number of vinculin clusters/area (**J**) for cells on glass (16.6 ± 2.1 N_cluster_/μm^2^) and graphene (18.0 ± 1.4 N_cluster_/μm^2^): the box shows 25/75th percentile and the whiskers are the standard deviation, T-test statistically non-significant. The total number of analyzed clusters is N_glass_ = 941 and N_graphene_ = 1147 for glass and graphene substrates, respectively, for *n* > 3 independent experiments. Kolmogorov–Smirnov Statistical test: ns nonsignificant, *** *p* < 0.001. Scale bar = 5 μm. Scale bar inset = 500 nm.

At DIV1 (Figure 3), we observed that neurons are indifferently located on glass and SLG. Super-resolution images showed the organization of vinculin on different substrates (Figure 3A,D), and we segmented the vinculin clusters (Figure 3B,E) with cluster analysis (Materials and Methods). Clustering provided the number of localized events per cluster and the cluster area distributions (Figure 3C,F) on the different substrates. On SLG substrates, we observed an 18% decrease in the number of vinculin localizations/cluster (Figure 3G) while the cluster area exhibited no significant differences (Figure 3H) compared to glass. The corresponding cluster density, calculated as the number of localizations divided by the cluster area, decreased (17%) accordingly (Figure 3I).

We next verified if the adhesion process could be mediated by an altered number of vinculin clusters per area. We measured the number of clusters per μm^2^, and the results show that no significant changes can be highlighted (Figure 3J). These results indicate an initial reduction of the vinculin distribution. Furthermore, one day in vitro is apparently too early to appreciate any recruitment/reorganization of new vinculin clusters that could mediate the neuronal adhesion on the substrates, consistent with the results reported by Lorenzoni and coworkers [28], which show equally distributed neurons on SLG and glass at the early stage after cell seedings (DIV1).

**Figure 4:**
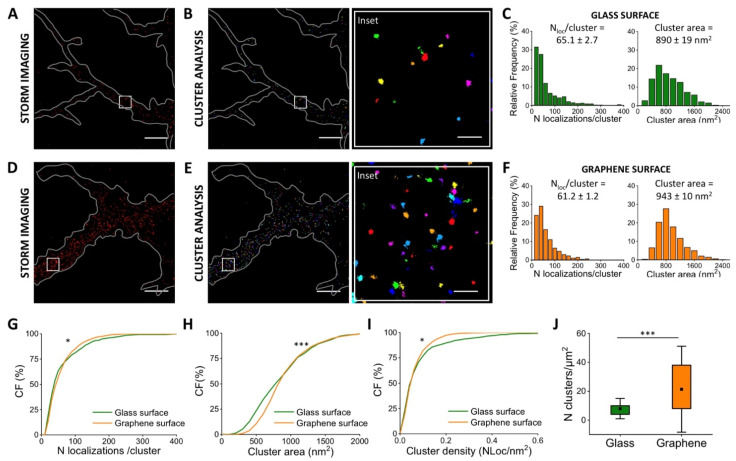
Quantitative super-resolution imaging of vinculin in neurons (DIV3). STORM imaging (**A**) and cluster analysis (**B**) of vinculin immuno-stained with A405/A647 in neurons seeded on glass. Frequency distribution of localizations/cluster and cluster area measured in cells seeded on glass (**C**). STORM imaging (**D**) and clustering (**E**) of vinculin immuno-stained with A405/A647 in neurons seeded on SLG. Frequency distribution of localizations and cluster area in cells grown on SLG (**F**). The cumulative distribution shows a 6% decrease in the average number of localized events (**G**) for vinculin in cells adhered on SLG (61.2 ± 1.2 N_loc_/cluster) compared to cells adhered on glass (65.1 ± 2.7 N_loc_/cluster). The cumulative distribution shows a 6% increase in the average cluster area (**H**) in cells adhered on SLG (943 ± 10 nm^2^) compared to cells adhered on glass (890 ± 19 nm^2^). The cumulative distribution shows a 23% decrease in average density (**I**) in cells adhered on SLG (0.072 ± 0.002 N_loc_/nm^2^) compared to cells adhered on glass (0.093 ± 0.005 N_loc_/nm^2^). The average number of vinculin clusters/area (**J**) increases in cells grown on graphene (21.0 ± 4 N_cluster_/μm^2^) compared to glass (8.0 ± 0.9 N_cluster_/μm^2^). The box shows 25/75th percentiles and the whiskers are the standard deviation, T-test statistically significant, *p* < 0.05. The total number of analyzed clusters is N_glass_ = 486 and N_graphene_ = 1275 for glass and graphene substrates, respectively, for *n* > 4 independent experiments. Kolmogorov–Smirnov Statistical test: ns nonsignificant, * *p* < 0.05, *** *p* < 0.001. Scale bar = 5 μm. Scale bar inset = 500 nm.

The same work also observed complete neuron migration on SLG after a few days in vitro [28]; we therefore extended our investigation to neurons at later growing stages (Figure 4). We performed STORM super-resolution imaging observing the vinculin distribution at three days in vitro (DIV3) both on glass (Figure 4A) and graphene (Figure 4D). As described for the neurons at DIV 1, the segmented vinculin clusters are identified (Figure 4B,E) and the localizations/cluster and the cluster area are quantified for the different substrates (Figure 4C,F). For cells grown on graphene, the number of localizations per cluster decreased (6%) compared to cells grown on the glass substrate (Figure 4G), while the average cluster area exhibits a 6% increase (Figure 4H). These values show a significantly decreased localization density (23%) when neurons are seeded on graphene (Figure 4I). These data seem to suggest a lower amount of vinculin expression, despite the preferential growth of neurons on the graphene substrate. To better understand the molecular mechanism behind the preferential adhesion of neurons on a given substrate, we measured the number of vinculin clusters per μm^2^. In this case, we observed a significantly higher number of vinculin clusters when cells are grown on graphene (Figure 4J). This result suggests a vinculin reorganization driven by the substrate, showing a highly fragmented vinculin distribution on graphene.

Although most individual vinculin clusters exhibit a lower density on the graphene substrate, we found a larger amount of the clusters per unit area. Importantly, comparing the number of clusters per unit area at DIV1 and at DIV3, we observed that the cluster density diminished on glass and increased on SLG (Figure 3J and Figure 4J). This fact could explain why neurons have been observed in previous investigations [22,28] to migrate from glass to SLG building the ordered neuronal network in the regions (i.e., SLG) where they become more tightly anchored.

Furthermore, the emergence of new vinculin clusters could be a key point for preferential adhesion, despite the reduced amount of vinculin within each cluster.

## 4. Discussion

Graphene has brought significant contributions to biomedical applications and neural tissue engineering due to outstanding properties, such as conductivity, mechanical strength, high surface area, and biocompatibility. Despite these promising attributes, it is becoming more and more important to characterize at the molecular level the interactions between the substrates and the biological samples. Still, the mechanisms behind the preferential adhesion of neurons and mammalian cells on the graphene substrates are not fully clear. It is well established that substrates functionalized with graphene-based materials are reportedly effective in regulating cellular microenvironments, affecting and enhancing key factors controlling various cell functions, which include adhesion, growth, and stem cell differentiation [7,48,49,50,51,52]. However, the exact mechanisms underneath would critically depend on the complex interplay of a variety of properties, including local roughness, polarity, and the amount of non-covalent interactions at the material surface that can influence protein concentration and consequently regulate cell adhesion. For this reason, the availability of highly reliable and quantitative methods to assess neuronal adhesion features on graphene systems is strongly desirable, since these can be critically important in elucidating surface-cell interaction, especially when these can be made at such a high spatial resolutions to be ultimately correlated to local substrate properties.

Here, we use single-molecule localization microscopy combined with cluster analysis to quantitatively investigate the distribution of the adhesion protein vinculin in CHO cells and neurons seeded on glass or graphene. The results have shown a different interaction of the cells on the two interfaces, consistent with our previous findings. In particular, CHO cells on SLG expressed a higher number of vinculin clusters, with larger size and containing a higher number of vinculin molecules as compared to glass, a finding in agreement with the higher detachment work from SLG previously measured by single cell force spectroscopy (SCFS). Neurons instead showed a re-arrangement and fragmentation of the vinculin clusters at increasing days in vitro; the density of clusters was significantly higher on SLG with respect to glass at DIV3. This finding nicely explains the migration of neurons toward SLG stripes observed previously on SLG micropatterned substrates [14,29].

Furthermore, our results demonstrate quantitative super-resolution as a suitable tool to investigate the interaction of cells with SLG, elucidating the molecular mechanisms behind their preferential adhesion and affinity. Optimized and efficient quantitative approaches, based on SMLM and cluster analysis, can be widely exploited in the future to characterize the cellular adhesion on bidimensional and other nanostructured material.

## 5. Materials and Methods

### 5.1. Single-Layer Graphene/Glass Substrate Fabrication

Commercially available CVD grown SLG on copper (Cu) (2-DTech, Cheltenham, UK) was transferred on glass coverslips by wet etching technique on Cu as following the protocol reported in [29]. The transferred SLG was ablated by laser micromachining following the protocol previously optimized and described by [28]. The result was a coverslip, half of SLG and half of glass. The substrates were coated with 0.1 mg/mL poly-D-lysine (PDL, Sigma-Aldrich, Sant Luois, MO, USA) for 3 h in an incubator at 37 °C and rinsed with sterile deionized water. The coverslip thickness was 18 mm (1.5 high precision, Marienfeld GmbH & Co, Lauda-Königshofen, Germany).

### 5.2. Cell Cultures

Chinese hamster ovary cell lines (ATCCs, CCL-61T, UK) were cultured under standard conditions in Dulbecco’s modified eagle medium (Gibco DMEM, Thermo Fisher Scientific, Waltham, MA, USA) until DIV2.

Primary hippocampal neurons were prepared from E18 rat as reported in Keshavan et al. [22]. Neurons were plated in serum-free Neurobasal-A medium (Invitrogen, Italy) supplemented with Glutamax (Invitrogen, Italy) 1%, B-27 (Invitrogen, Italy) 2%, at 37 °C in 5% CO_2_ until DIV1 or DIV3.

### 5.3. Immunostaining Protocol

CHO Cells were fixed with 3% (*w*/*v*) paraformaldehyde (PFA, Sigma-Aldrich, Sant Luois, MO, USA) in phosphate-buffered saline (PBS, pH 7.4 Thermo Fisher Scientific, Waltham, USA) for 7 min at room temperature (RT). Embryonal rat hippocampal neurons were fixed with 4% (*w*/*v*) paraformaldehyde (PFA) in phosphate-buffered saline (PBS) for 30 min at 22 °C temperature (RT).

After washing 3 times in PBS, cells were incubated with a blocking buffer solution containing 3% (*w*/*v*) bovine serum albumin (BSA, Sigma-Aldrich) and 0.2% Triton X-100 (Sigma-Aldrich) for 40 min at RT to prevent non-specific binding and permeabilize the cell membrane.

Immunolabeling of vinculin was performed incubating cells with an anti-vinculin rabbit primary antibody (PA5-19842, Thermo Fisher, Waltham, MA, USA) at RT for 1 h, followed by 45 min incubation with a custom-built anti-rabbit secondary antibody conjugated with the dye pair Alexa Fluor 405/Alexa Fluor 647 (conjugation protocol in the following paragraph) at RT. At the end of the immunostaining, samples were fixed again in PFA 2% for 5 min and stored in PBS at 4 °C.

### 5.4. Activator-Reporter Dye Pairs Preparation Protocol

For STORM imaging, the photo-switchable secondary antibody consisting of a dye activator/reporter was custom prepared following the STORM-protocol sample preparation [53].

Briefly, secondary antibody used was a donkey anti-rabbit from Jackson ImmunoResearch Europe. The dyes were purchased as NHS ester derivatives: Alexa Fluor 405 carboxylic acid succinimidyl ester (Invitrogen), and Alexa Fluor 647 carboxylic acid succinimidyl ester (Invitrogen). Antibody labeling reaction was performed by incubating, for 40 min at RT, a mixture containing the secondary antibody, NaHCO3, and the appropriate pair of activator/reporter dyes diluted in dimethyl sulfoxide, anhydrous (DMSO) (Sigma-Aldrich).

Purification of labeled antibody was performed using NAP5 Columns (GE HealthCare).

### 5.5. STORM Imaging and Data Reconstruction

#### 5.5.1. STORM Microscope

A commercial N-STORM TIRF microscope (Nikon Europe BV, Amsterdam, Netherlands,), equipped with an oil immersion objective (CFI Apo TIRF 100x, NA 1.49), was used to acquire 20,000 frames at a 33 Hz frame rate using highly inclined illumination. The duration of the acquisition was the same in all experiments.

An excitation intensity of ~1.0 kW/cm^2^ for the 647 nm read-out (300 mW laser; MPB Communications, Pointe-Claire, QC, Canada) and an activation intensity of ~30 W/cm^2^ (100 mW laser; Coherent, Santa Clara, CA, USA) were used. A repeating cycle of 1 activation frame followed by 3 read-out frames was used, and imaging was performed with an EMCCD camera (Andor iXon DU-897, Andor Technologies, Belfast, UK). The Nikon Perfect Focus System was applied during the entire recording process. Fluorescence-emitted signal was spectrally selected by the four colors dichroic mirrors (ZET405/488/561/647; Chroma Technology Corp., VT, USA) and filtered by a multiband pass filter (ZT405/488/561/647; Chroma).

#### 5.5.2. Imaging Buffer

All samples were imaged in the previously described GLOX imaging buffer, containing a glucose oxidase solution as the oxygen scavenging system (40 mg/mL^−1^ catalase (Sigma-Aldrich), 0.5 mg/mL^−1^ glucose oxidase, 10% glucose in PBS) and MEA 10 mM (cysteamine MEA (#30070-50G; Sigma-Aldrich) in 360 mM Tris-HCl) [53].

#### 5.5.3. Imaging Protocol

Imaging was performed by acquiring 20,000 frames of 647 channel with an exposure time of 30 ms. The 647 nm laser was used for exciting the reporter dye (Alexa 647) and switching it to the dark state. The 405 nm laser light was used for reactivating the reporter into a fluorescent state via the activator dye (Alexa 405). An imaging cycle was used in which one frame belonging to the activating light pulse was alternated with three frames belonging to the imaging light pulse.

#### 5.5.4. Analysis of Raw STORM Data

Image reconstruction was performed using a custom software (Insight3, kindly provided by Dr. Bo Huang of the University of California) by Gaussian fitting of the single-molecule images in each frame to determine the x–y coordinates. The molecules were identified by always setting the same threshold of counts/pixel. The final images were obtained by plotting each identified molecule as a Gaussian spot and corrected for drift by cross correlating images obtained from subsets of frames as described in the literature [54].

#### 5.5.5. Cluster Analysis

Cluster analysis of localized STORM data was performed with a MATLAB (The MathWorks, Natick, MA, USA) custom-written code implementing a distance-based clustering algorithm [47]. The code belongs to the density-based clustering family and identifies spatial clusters of localizations. It is suitable for analyzing both high-density and low-density protein distributions optimally because it allows acting on a scale factor that determines the segmentation degree of examined clusters without affecting the clustering ability of the algorithm.

First, the localization lists are binned to construct discrete localization images with a pixel size of 20 nm. These were filtered with a square kernel (7 × 7 pixel^2^) to obtain a density map and transformed into binary images by applying a constant threshold, such that pixels have a value of 1 where the density is larger than the threshold (and 0 elsewhere). These binary images were used only to locate regions of the sample containing localizations. Further analyses were performed on raw localization data. Only localizations lying on adjacent (six-connected neighbors) nonzero pixels of the binary image were considered from the binary images. Localization coordinates within each connected component were grouped employing the distance-based clustering algorithm. Initialization values for the number of clusters and the relative centroid coordinates were obtained from the local maxima of the density map within the connected region, and localizations were associated with clusters based on their proximity to cluster centroids. New cluster centroid coordinates were iteratively calculated as the average of localization coordinates belonging to the same cluster. The procedure was iterated until convergence of the sum of the squared distances between localizations and the associated cluster [47].

The algorithm relies on a limited number of parameters and allows setting a factor, whose value determines the degree of segmentation of clusters, and a threshold of minimum number of molecules, in order to avoid noise. Before analyzing localization data, we optimized clustering factors and parameters to obtain the best performance of the algorithm on vinculin. Image analysis was performed with the same selected parameters for all the measurements.

The algorithm provided cluster centroid positions and the number of localizations per cluster.

### 5.6. DNA Origami Preparation, Deposition and Attachment on Substrates

DNA origami preparation. DNA origami structures with 86 binding site “handles” for fluorophore modified DNA oligonucleotides (“anti-handles”) were prepared in 50 ul volumes as described in [46]. Scaffold strands (10 nM) were mixed with 100 nM staple strands in 0.5X TBE supplemented with 12.5 mM MgCl_2_ to form the origami structure. Additionally, 19.35 µM of Atto647N modified anti-handles were included for fluorescent labeling and 150 nM biotinylated anti-handles were included for facilitating surface attachment to the substrates. Mixtures were heated to 65C and then cooled incrementally over 1 h to 4C. Structures were then purified using glycerol gradient centrifugation [55].

DNA origami structures on glass: Wells of a µ-Slide 8 Well plate Glass Bottom (Ibidi GmbH, Germany, #80827) were rinsed 3× with MilliQ water, washed for 5 min at RT with 1M NaOH, and again rinsed 3× with MilliQ water. The surface of the wells was coated with Poly-L-Lysine for 30 min at RT and rinsed 3× with MilliQ water. Next, a biotin solution of 0.5 mg/mL in water was added for 5 min (volume of 120 µL) and subsequently washed 3× with MilliQ water. Streptavidin solution of 0.5 mg/mL in water was added next for 5 min at RT (volume of 120 µL) and rinsed again 3× with MilliQ water. The concentration of DNA origami, measured by nanodrop spectrophotometer, was about 3.8 nM.

For the attachment of DNA origami to the glass, a solution (1:15) of DNA origami in 10 mM MgCl_2_ was used (a droplet of 80 µL in the center of the well); time of attachment 10 min at RT. After this time the modified imaging buffer was used (with addition of MgCl_2_): 395 µL of PBS + 5 µL of 1M MgCl_2_, 40 µL of glucose, 40 µL of MEA, and 4 µL of glucose oxidase (GLOX, Sigma-Aldrich, Sant Luois, MO, USA).

DNA origami structures on graphene: Wells of a µ-Slide 8 Well plate Glass Bottom (Ibidi GmbH, Germany, #80827) were rinsed 3× with MilliQ water and coated with Poly-L-Lysine for 30 min at RT and rinsed again 3× with MilliQ water. DNA origami solution (1:15) in 10 mM MgCl_2_ was added directly on a coated graphene surface (similarly as in the case of attachment to the glass surface). After the attachment, the surface was rinsed gently with 10 mM MgCl_2_, and the modified imaging buffer was used (as above).

## 6. Statistical Analysis

All statistical tests were performed in OriginPro2019. Data corresponding to the number of localizations, cluster area, and density were tested with a non-parametric Kolmogorov–Smirnov test. Similarly, data corresponding to the number of clusters per area were tested with unpaired two-tailed Student’s *t*-test. *p*-values were considered significant if <0.05. Bar graphs are displayed as mean ± standard error of the mean (SEM) unless otherwise noted. The box size indicates 25/75th percentiles, and the whiskers correspond to the standard deviation unless otherwise specified.

## Data Availability

The data presented in this study are available in FigShare at 10.6084/m9.figshare.16999159.

## Supplementary Materials

The following are available online at https://www.mdpi.com/article/10.3390/membranes11110878/s1, Figure S1: “Effect of the graphene substrate on single molecule localization events measured on DNA origami nanostructures”.
