# Supplementary material for: Quantitative Super-Resolution Microscopy to Assess Adhesion of Neuronal Cells on Single-Layer Graphene Substrates
Source: Membranes (Basel). 2021 Nov 15;11(11):878. doi: 10.3390/membranes11110878 (PMC8621106; doi:10.3390/membranes11110878)
Supplement: Supplementary file 1 [file membranes-11-00878-s001.zip › membranes-1452201-supplementary.pdf]

SUPPLEMENTARY INFORMATION:

Quantitative super-resolution microscopy to assess adhesion of neuronal cells on single-layer graphene substrates

Silvia Scalisi[1,4,5], Francesca Pennacchietti[1], Sandeep Keshavan[2], N. D. Derr[3], Alberto Diaspro[1, 4], Dario Pisignano[6,7], Agnieszka Pierzynska-Mach[1], Silvia Dante[2,*], Francesca Cella Zanacchi[6,1,*]

Supplementary material: The following are available online, Figure S1: Effect of substrate on single molecule localization experiments measured on DNA origami nanostructures.

Supplementary Figure S1

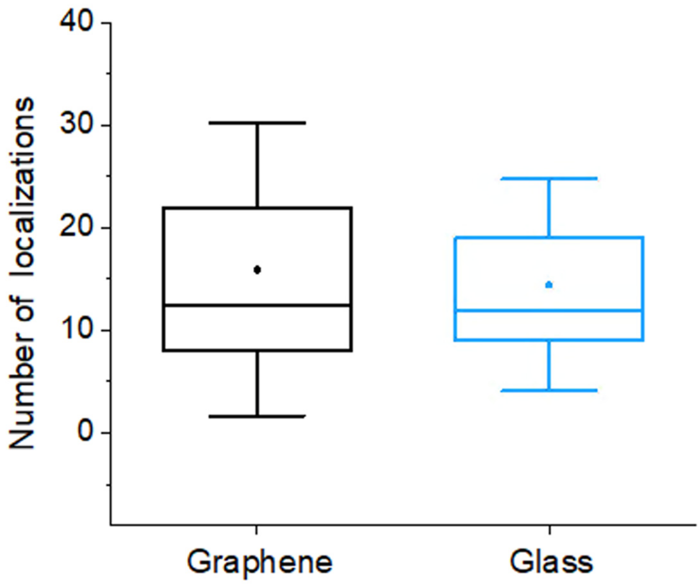

Supplementary Figure S1 **Effect of the graphene substrate on single molecule localization events measured on DNA origami nanostructures.** Number of localizations collected on DNA origami with 86 handles functionalized with Atto640 fluorophores and absorbed on glass and graphene substrates. The blinking events localized are not affected by the graphene substrate coated with poly-L-lysine. Despite the larger variability and standard deviation exhibited on the graphene surface, DNA origami structures show a comparable value for the number of localizations/structure on graphene (16±10 Nloc/structure) and on glass substrates (14±7 Nloc/structure). The total number of analyzed clusters is $N_{graphene}$=102 and N_{glass}=339 for glass and graphene substrates, respectively. n>3 independent experiments. Line: median, Dot: mean, Whiskers: standard deviation.
Kolmogorov-Smirnov Statistical test: ns nonsignificant.
